# Head-to-Head Comparison of UHPLC-MS/MS and Alinity C for Plasma Analysis of Risperidone and Paliperidone

**DOI:** 10.3390/ph17111446

**Published:** 2024-10-29

**Authors:** Francisco José Toja-Camba, Gonzalo Hermelo-Vidal, Carolina Feitosa-Medeiros, María Vidal-Millares, María José Durán-Maseda, Anxo Fernández-Ferreiro, Cristina Mondelo-García

**Affiliations:** 1 Pharmacy Department, University Clinical Hospital of Santiago de Compostela (SERGAS), 15706 Santiago de Compostela, Spain; kikotoja@gmail.com; 2 FarmaCHUSLab Group, Health Research Institute of Santiago de Compostela (IDIS), 15706 Santiago de Compostela, Spain; zalohermelo@gmail.com (G.H.-V.); carolinafeimed@gmail.com (C.F.-M.); 3 Faculty of Pharmacy, University of Santiago de Compostela, 15782 Santiago de Compostela, Spain; 4 Psychiatry Department, University Clinical Hospital of Santiago de Compostela (SERGAS), 15706 Santiago de Compostela, Spain; maria.vidal.millares@sergas.es (M.V.-M.); maria.jose.duran.maseda@sergas.es (M.J.D.-M.)

**Keywords:** risperidone, paliperidone, UHPLC-MS/MS, Alinity C, pharmacokinetics, TDM

## Abstract

**Background and objectives:** Risperidone, a second-generation antipsychotic widely used in the treatment of schizophrenia, requires therapeutic drug monitoring due to its high interindividual variability. UHPLC-MS/MS is considered the gold standard for pharmacokinetic studies owing to its superior sensitivity and specificity, although it involves time-consuming manual sample preparation. In contrast, the Alinity C system, fully automated, simplifies sample processing, but only measures the active moiety (risperidone plus paliperidone). The aim of this study is to compare the performance of UHPLC-MS/MS and the Alinity C system for the determination of risperidone and paliperidone concentrations in plasma. **Methods:** A total of 115 plasma samples of 115 patients, 92 and 23 under risperidone and paliperidone long-acting treatment, respectively, were analyzed using both methods. **Results:** A strong correlation for the active moiety (risperidone plus 9-OH-Risperidone) (rs = 0.95) was observed. However, Bland–Altman analysis revealed a mean bias of 0.996 ng/mL, indicating that the Alinity C system slightly overestimates concentrations compared to UHPLC-MS/MS. While there was substantial agreement between methods (κ = 0.72), discrepancies were observed in 16.3% of cases, which could impact clinical decision-making. When analyzing paliperidone separately, the agreement was lower (κ = 0.63), with greater variability observed. **Conclusions:** These findings suggest that, while the Alinity C system is suitable for routine therapeutic monitoring, UHPLC-MS/MS remains the preferred method in clinical scenarios requiring higher precision, particularly for patients with concentrations near therapeutic thresholds.

## 1. Introduction

Mental disorders constitute an important health system problem due to their high complexity and their major impact on the quality of life of patients [1]. Specifically, schizophrenia is a chronic brain disorder which presents both positive and negative symptoms affecting cognition and emotions [2].

The pharmacological treatment of schizophrenia is based on antipsychotics. These drugs exhibit a high intra- and inter-individual variability and they are often less effective than expected [3]. Among them, risperidone is a second-generation drug which has proven efficacy in treating psychotic and affective symptoms [4]. In cases of treatment resistance, dose optimization needs to be performed based on trial and error, as the therapeutic effect cannot currently be predicted. Together with a poor insight into the disease, severe and frequent side effects are the main reasons for discontinuation of treatment [5,6].

In clinical practice, deviations from the recommended dose stated in the summary of product characteristics are often needed to provide a sufficient clinical response with minimal side effects. It is very difficult for doctors to determine an optimum dose without having objective information, for example, plasma drug concentrations, due to tremendous variability between individuals. Therefore, such an empirical method may postpone the attainment of treatment objectives and can even cause relapses or complications, which are bad prognostic signs, as well as low adherence to treatment [7].

Risperidone is metabolized through cytochrome CYP2D6 to its main metabolite, paliperidone. The therapeutic reference range trough values were reported by the Therapeutic Drug Monitoring (TDM) task force of the AGNP consensus to be between 20 and 60 ng/mL for the active moiety (risperidone plus 9-OH-Risperidone) and for paliperidone individually [8]. In this sense, according to Hart et al. [9], dose optimisation after initial prescription or dose change of risperidone is indicated to attain therapeutically recommended target concentrations associated high efficacy and acceptable tolerability.

In the last several years, a growing number of authors have stressed the role of personalized medicine in psychiatric care. The concept of precision psychiatry has drawn a line under the need for highly informative analytical methods that would provide accurate and reliable data to support decision-making in clinical practice [10]. In this regard, adequate plasma concentration measurement is required to achieve optimal risperidone therapy outcomes. Therefore, risperidone quantification is very important for individualizing treatment to meet specific needs of patients who may be under this medication to avoid adverse reactions and enhance therapeutic effects [11]. 

Several chromatographic techniques have been described for risperidone analysis. Specifically, Ultra-high-performance liquid chromatography–tandem mass spectrometry (UHPLC-MS/MS) has emerged as a robust tool from the analytical viewpoint, providing high sensitivity and selectivity towards compound quantification in biological samples. Discriminating between risperidone and paliperidone via mass spectrometry makes it possible to detect them with precision and specificity, thus responding to the need for accurate TDM for psychiatric inpatients. 

The UHPLC-MS/MS method is known to be the gold standard in pharmacokinetic studies due to its unrivalled sensitivity and selectivity [12]. Therefore, its capacity to quantify analytes at lower concentration levels without interference from matrix components or related compounds makes it well suited for successful analysis during investigations into risperidone and its metabolite in complex biological matrices. Sample preparation before analysis is entirely manual; this could bring potential variability and it is very time-consuming [13].

On the other hand, an attractive alternative analytical method used habitually in clinical practice is the Alinity C system (Abbot Laboratories). It uses photometry and potentiometry and is technically feasible for point-of-care testing due to its fully automated nature with ease of handling. The degree of automation is one of the hallmarks of the Alinity C system that decreases variability due to manual sample preparation steps. Such features meet the increased demands today placed on efficiency attributes of high-performance methods in clinical diagnostic laboratories where high-throughput capacity aspects take precedence [13]. Currently, there are no comparative studies between the gold standard and Alinity C; consequently, this study is highly relevant to ensure adequate reliability of the results provided in clinical practice.

This is the first study that compares the performance of these two analytical methods for the plasma determination of risperidone and paliperidone: UHPLC-MS/MS and the Alinity C system.

## 2. Results

### 2.1. UHPLC-MS/MS vs. Alinity C—Active Moiety

The active moiety concentrations of 92 samples from 92 patients under risperidone long-acting treatment were determined by both methods. Median values of 39.33 ng/mL (IQR: 23.36–59.38) for UHPLC-MS/MS and 43 ng/mL (IQR: 25–61) for Alinity C were obtained. The mean concentrations determined for UHPLC-MS/MS and Alinity C were 43.88 ng/mL (SD: 23.34) and 44.88 ng/mL (SD: 22.20), respectively (Table 1). The Spearman rank correlation coefficient showed a good correlation between both methods with a value rs = 0.95.

#### 2.1.1. Bland–Altman and Regression Analysis—Active Moiety

**Figure 1:**
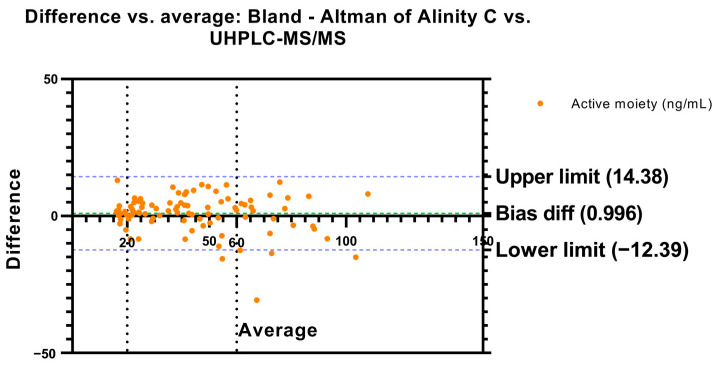
Bland–Altman’s plot. Difference in active moiety concentrations (risperidone plus paliperidone) vs. the average between Alinity C and UHPLC-MS/MS. Dashed green line represents the bias difference and dashed blue lines represent the limits of agreement. Risperidone therapeutic reference range is represented by dashed black lines.

**Figure 2:**
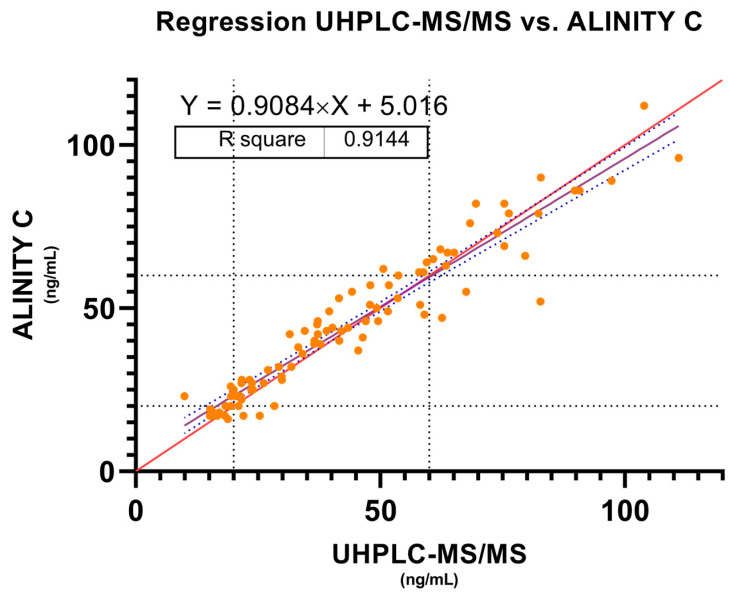
Passing–Bablock regression analysis. Measured concentrations of active moiety between Alinity C and UHPLC-MS/MS. Red line represents the identity line; blue dashed lines represent the 95% confidence bounds and risperidone therapeutic reference range is represented by dashed black lines.

To determine the degree of agreement between the two methods in measuring active moiety concentrations, a Bland–Altman analysis was performed. The mean difference, or bias, between the two methods was 0.996 ng/mL, while the standard deviation of the error was 6.829. The confidence interval established at 95% was also calculated using the Bland–Altman method, showing an upper limit of 14.38 and a lower limit of −12.39, establishing an interval where the differences obtained between the two methods were found in 95% of the cases (Figure 1). The results obtained in this Bland–Altman analysis were reinforced by performing a regression analysis in which a slope of 0.908 and an intercept of 5.016 with a correlation coefficient (r = 0.91) were found (Figure 2). A Bland–Altman percent difference analysis was also performed and is available in the Supplementary Material (Figure S1). 

#### 2.1.2. Stratification in Ranges—Active Moiety

Active moiety concentrations were classified according to the range in which they fell with respect to the therapeutic reference range (<20 ng/mL: sub-therapeutic; 20–60 ng/mL: therapeutic range; >60: supra-therapeutic) (Table 2). Cohen’s Weighted Kappa was calculated, which showed substantial agreement between the measurements obtained by both methods (K = 0.72 ± 0.06). The agreement between methods taking into account all samples was 83.69%. Specifically, 8/10 (80%) samples in the sub-therapeutic range, 51/59 (86.44%) in the therapeutic range and 18/23 (78.26%) in the supra-therapeutic range. Therefore, the clinical decision to be taken could be different in 16.3% of the cases. Finally, McNemar’s test was used to determine whether there are discordances between the different cut-off points (sub-therapeutic/therapeutic range and therapeutic range/supra-therapeutic range). No significant differences were found (*p* = 0.453 and *p* = 0.7266).

Taking into account that Alinity C does not provide data on the concentrations of risperidone and its active metabolite, 9-OH-R, but only on the active moiety, we classified the samples according to the paliperidone/risperidone ratio and used the differences found between both methods to compare whether there were differences that could be influenced by the excess or deficiency of any of the analytes. For this purpose, we performed an ANOVA test that showed no significant differences (*p* = 0.92) between the seven groups compared.

### 2.2. UHPLC-MS/MS vs. Alinity C—Paliperidone

#### 2.2.1. Bland–Altman and Regression Analysis—Paliperidone

**Figure 3:**
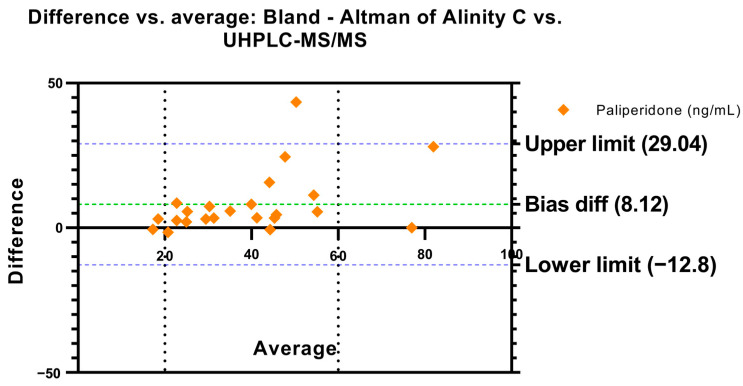
Bland–Altman’s plot. Difference in paliperidone concentrations vs. the average between Alinity C and UHPLC-MS/MS. Dashed green line represents the bias difference and dashed blue lines represent the limits of agreement. Paliperidone therapeutic reference range is represented by dashed black lines.

**Figure 4:**
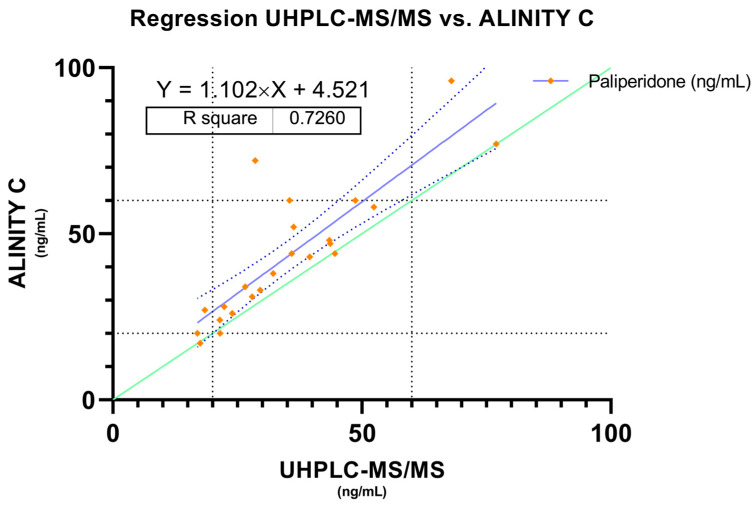
Passing–Bablock regression analysis. Measured concentrations of paliperidone between Alinity C and UHPLC-MS/MS. Green line represents the identity line, blue dashed lines represent de 95% confidence bounds and risperidone therapeutic reference range is represented by dashed black lines. Paliperidone therapeutic reference range is represented by dashed black lines.

A total of 23 samples from 23 patients treated with long-acting paliperidone were analyzed in parallel, in order to check if both methods are comparable also in the measurement of this particular analyte. A Bland–Altman analysis was also performed which showed a mean error of 8.12 ng/mL, the confidence interval was set at 95% with an upper limit of 29.04 ng/mL and a lower limit of −12.8 ng/mL (Figure 3). In this case, a regression analysis was also performed in which a slope of 1.10 and an intercept of 4.52 with a correlation of r = 0.72 were found (Figure 4). Also, a Bland–Altman percent difference analysis was performed and is available in the Supplementary Material (Figure S2).

#### 2.2.2. Stratification in Ranges—Paliperidone

In the same way as for the active moiety, the results were stratified according to the different ranges (Table 3). In this case the Cohen’s Weighted Kappa also revealed substantial agreement (K = 0.63 ± 0.18) between the different cut-off points. A McNemar test was also performed in which no statistically significant differences were obtained between the two cut-off points analyzed (*p* = 0.5 and *p* = 1).

## 3. Discussion

The field of psychiatric pharmacotherapy requires precise accurate analytical methodologies which are capable of providing personalized treatment options. In this sense, the choice between analytical methods extends beyond technical considerations to encompass practical aspects, such as cost, ease of use and scalability [14]. To the best of our knowledge, this is the first study comparing a UHPLC-MS/MS system with an Alinity C system for assaying risperidone and paliperidone in plasma.

UHPLC-MS/MS is considered the gold standard in pharmacokinetic studies but features manual sample processing, which may introduce variability and be time consuming. Moreover, its high sensitivity and specificity in distinguishing closely related compounds make it valuable for research purposes [12]. The Alinity C system, in contrast, features automation that reduces the analytical variability related to manual sample processing. Consequently, it is practical for routine clinical measurements but provides values for the active moiety and not separately for each component. 

In the Bland–Altman analysis, a positive bias of 0.996 was observed, indicating that, on average, the Alinity C method reports slightly higher values compared to the UHPLC-MS/MS method. This suggests that the automated Alinity C method tends to overestimate concentrations compared to the reference method, as has been observed by the authors in another comparison between these two methods for aripiprazole [13]. In addition, the 95% limits of agreement, ranging from −12.39 to 14.38, indicate moderate variability in the difference between the two methods, reflecting that, although there is a general tendency to overestimate, the differences between the methods can be wide in some particular cases. This variability is consistent with the standard deviation of the bias of 6.829.

The linear regression between the two methods, on the other hand, showed a slope of 0.9084 (95% CI 0.8502 to 0.9666), indicating a strong, but not perfect, relationship between the two methods. A slope less than 1 suggests that as UHPLC-MS/MS values increase, Alinity C values grow at a slower rate, which is consistent with the positive bias observed in Bland–Altman. Furthermore, the Y-intercept of 5.016 (95% CI: 2.127 to 7.906) reinforces the existence of a systematic difference between the two methods. The coefficient of determination (R^2^ = 0.9144) shows that the Alinity C method explains more than 91% of the variability in the UHPLC-MS/MS measurements, indicating good agreement despite systematic differences.

The data obtained by classifying the concentrations of the active moiety into therapeutic ranges (<20 ng/mL, 20–60 ng/mL, >60 ng/mL) show good agreement between Alinity C and UHPLC-MS/MS, as reflected by Cohen’s Weighted Kappa (0.72 ± 0.06) [15]. This is consistent with the high coefficient of determination (R^2^ = 0.9144), suggesting that, in clinical practice, both methods provide similar results in most cases. However, the difference observed in 16.3% of the samples could have clinical implications, as it could lead to different therapeutic decisions. Despite these differences, the results of McNemar’s test revealed no significant differences between the cut-off points, suggesting that the two methods do not show a systematic tendency to misclassify at key clinical decision thresholds.

The lack of significant differences observed in the ANOVA between the different groups stratified by paliperidone/risperidone ratio is also consistent with the overall results, which reinforces the idea that the discrepancies between Alinity C and UHPLC-MS/MS are not due to variations in analyte concentrations. This is significant because the paliperidone/risperidone ratio does not stay consistent over time or across different patients, particularly in those with CYP2D6 metabolizing phenotypes that differ from the normal phenotype [4]. This suggests that the bias observed in the Bland–Altman analysis (bias of 0.996) is likely due to methodological differences between the measurement platforms and not due to misrepresentation of the individual components of the active moiety. Despite these differences, the overall results show that Alinity C is a useful method for therapeutic classification in most cases, although the possibility of discrepancies in patients close to therapeutic cut-off points should be considered.

In the case of the determination of paliperidone, higher methodological differences were observed compared to the results obtained for the active moiety. The Bland–Altman analysis revealed a higher bias (8.120) and wider limits of agreement (−12.80 to 29.04), indicating greater variability in the differences between methods. Although the slope of the regression (1.102) suggests that Alinity C tends to slightly overestimate paliperidone concentrations relative to UHPLC-MS/MS, the coefficient of determination (R^2^ = 0.7260) is lower compared to active moiety, reflecting lower agreement between methods when measuring paliperidone alone. This translates into greater scatter in the data, which is consistent with the higher standard deviation of the bias (10.67 vs. 6.829 in the active moiety analysis). However, no statistically significant differences were found in therapeutic cut-off points by McNemar’s test or Weighted Kappa (K = 0.63), which suggests moderate agreement between methods.

In regard to real-life clinical application, choosing between UHPLC-MS/MS and the Alinity C system should be based on the distinctive needs of the clinical setting. While UHPLC-MS/MS retains its place in research and special cases demanding the utmost sensitivity and specificity, the automation of the Alinity C system suggests it as a very good option for routine monitoring of therapeutic drugs, as it provides efficiency without precision loss [16].

When stratifying paliperidone concentrations into therapeutic ranges, agreement was lower than for the active moiety, particularly for samples within the supra-therapeutic range, where there was agreement in the 66% of the samples. These results suggest that the ability of Alinity C to accurately measure paliperidone is more limited at the extremes of the therapeutic ranges, which could have important clinical implications for patient dosing. Taken together, these findings for both the active moiety and paliperidone reinforce the idea that Alinity C provides useful results with good agreement in most cases, but its accuracy decreases when measuring paliperidone exclusively or in patients near therapeutic thresholds. This lower concordance for paliperidone suggests that, in clinical situations requiring more accurate monitoring of this particular metabolite, the use of UHPLC-MS/MS remains preferable, especially when clinical decisions depend on concentrations near the limits of the therapeutic range.

Regarding the evaluation of the comparability of UHPLC-MS/MS and Alinity C methods, the main limitation of this study is the sample size for paliperidone, which might not be large enough to generalize the findings to a wider population. This should encourage further research that probes possible sources of disparity in the future, including the presence of interfering substances or method-specific limitations.

Nevertheless, it is important to recognize the limitations of each method and carefully evaluate the special requirements of different clinical settings. While systematic method validation should be considered, the Alinity C system offers an alternative with its automation and ease-of-use advantages, making it very suitable for routine clinical implementations. Nonetheless, for clinical situations that demand higher precision, particularly when measuring paliperidone alone or in patients near therapeutic thresholds, UHPLC-MS/MS remains the preferred method.

## 4. Materials and Methods

A retrospective study was proposed, where plasma samples of patients treated with risperidone and paliperidone were selected. The study and data collection strictly adhered to the principles of the Declaration of Helsinki. Ethical approval was given by the local Institutional Review Board and the autonomous region of Galicia (2021/285).

### 4.1. UHPLC-MS/MS

Concentrations of risperidone and paliperidone in plasma were measured by a validated UHPLC-MS/MS method on a Xevo TQD^®^ triple quadrupole mass spectrometer (Waters, Milford, MA, USA). Risperidone-d4 was used as the internal standard. Risperidone, paliperidone and risperidone-d4 detection were monitored at the transition pairs *m*/*z* 441.2 → 191.1, *m*/*z* 427.24 → 207.05 and *m*/*z* 415.2 → 195.1, respectively.

For this purpose, 475 μL of plasma with 50 μL of risperidone-d4 at a concentration of 2 mg/L was added in duplicate to an Eppendorf and shaken by vortex-mixing for 1 min. These two samples were extracted from this solution, each consisting of 200 μL of the combined solution and 500 μL of acetonitrile (Fisher Chemicals, Zurich, Switzerland). Subsequently, they were vortex-mixed for 1 min and centrifuged (3500 rpm, 10 min, 4 °C), and 500 μL of the resulting supernatant was extracted. Then, the samples were evaporated to dryness and finally dissolved in 500 μL of a solution composed of water and acetonitrile, in a ratio of 85:15. 

A linear calibration curve was obtained over a concentration range of 1–200 ng/mL (R^2^ = 0.99). The detection and quantification limits for both risperidone and paliperidone were 0.5 and 1 ng/mL, respectively. An ACQUITY UHPLC BEH Shield RP18 column (2.1 × 50.0 mm, 1.7 μm) was used at a temperature of 40 °C. The mobile phase was a mixture of 10 mM ammonium adjusted to pH = 3 with formic acid (Fisher Chemicals, Zurich, Switzerland) (Mobile Phase A), in gradient mode with acetonitrile acid (Fisher Chemicals, Zurich, Switzerland) (Mobile Phase B), as shown in Table 4.

The sample manager was set to 4 °C, the flow rate to 0.6 mL/min and the injected volume of sample to 3 μL. The obtained data were processed using MassLynx^®^ software (Version 4.20.0001).

### 4.2. Alinity C

Analyzers such as Alinity C implement photometric and potentiometric detection technologies for the quantitative measurement of analytes in human serum, plasma, urine, cerebrospinal fluid, hemolysate and whole blood. The photometric assay for the active moiety of risperidone on the Alinity C Analyzer uses a tungsten lamp, diffraction grating and silicon photodiode detector. The measurement was taken at a wavelength of 604 nm. An assay for total risperidone in whole blood was developed, validated and tested for cross-reactions by Saladax Biomedical using the MyCare Psychiatry Kit (Saladax Biomedical, Bethlehem, PA, USA). The immunoassay is based on competition between the analyte in the blood specimen from the individual taking medication and calibrators, controls, and quality control materials provided in the kit for binding to risperidone-specific antibodies covalently bound to gold nanoparticles. Spectrophotometric measurements can be tracked using clinical chemistry analyzers such as Alinity C. Cross-reactivity testing was performed by the manufacturer with over 150 drugs and with the presence of interfering substances like rheumatoid factor, lipids, and hemolyzed blood. The lower limit of quantification of the kit is 16 ng/mL, and the upper limit of quantification is 120 ng/mL.

As Alinity C only provides the values of active moiety (risperidone plus paliperidone), the sum of drug and active metabolite found by UHPLC MS/MS has been used for the comparison between both methods.

A total of 115 plasma samples, corresponding to trough concentrations from 92 patients under risperidone long-acting treatment and 23 patients under paliperidone long-acting treatment, were collected. Samples were stored at −80 °C until analysis. Samples were analyzed by both UHPLC-MS/MS and Alinity C. Due to the clinical repercussions of possible variations in the use of one method or another, the samples have been stratified according to the consensus therapeutic range into infra-therapeutic (<20 ng/mL), therapeutic (20–60 ng/mL) and supra-therapeutic (>60 ng/mL). Furthermore, it was studied whether there were differences between the two methods when the paliperidone/risperidone ratios were different, and thus whether the amount of each analyte individually affected the determination by Alinity C. For this purpose, the proportion between the concentrations measured by the two methods (UHPLC-MS/MS/Alinity C) was classified into 7 different groups according to the paliperidone/risperidone ratio (<1, 2–3, 3–4, 4–5, 5–6, >6).

### 4.3. Statistics

Normality was tested using the Shapiro–Wilk test, and correlations were assessed using the Spearman rank correlation coefficient (rs). The Wilcoxon rank sum test was used to determine differences between drug levels. Bland–Altman analysis was performed to assess the agreement between the two quantitative measurements. A Bland–Altman plot shows the difference (y-axis) between these two measurements. The mean (x-axis) represents the average difference between two measurements, and the limits of agreement are expressed as the mean difference plus or minus 2 standard deviations of the difference. Additionally, Passing–Bablock regression was performed to estimate the best-fit line by comparing observed ranks between concentrations measured by UHPLC-MS/MS and Alinity C for active moiety and paliperidone.

Agreement between classification of UHPLC-MS/MS and Alinity C into three different therapeutic range groups was determined using Cohen’s Weighted Kappa(κ), and differences in classification for each group were assessed using McNemar’s test. To assess if the presence of higher concentrations of risperidone or its active metabolite paliperidone affect the active moiety determined by the Alinity C system, an ANOVA test was performed to determine differences between group means. 

## 5. Conclusions

This study offers insightful information about comparing UHPLC-MS/MS results with those of the Alinity C system regarding plasma concentrations of risperidone and paliperidone. Both methods showed high precision and substantial agreement at therapeutic ranges, except in the measurement of paliperidone, for which Alinity C appears to perform worse. These results confirm that the Alinity C system is justified in practical laboratory applications by its capacity to improve effectiveness and its consistency in therapeutic drug monitoring.

## Figures and Tables

**Table 1:** Descriptive statistics of UHPLC-MS/MS and Alinity C assays.

	UHPLC-MS/MS Risperidone	UHPLC-MS/MS Paliperidone	UHPLC-MS/MS Active Moiety	Alinity C Active Moiety	Ratio Paliperidone/ Risperidone	UHPLC-MS/MS Paliperidone	Alinity C Paliperidone
Samples	92	92	92	92	92	23	23
Minimum	0.79	2.53	9.95	16	0.07	16.96	17
Maximum	67.22	82.03	111	112	79.84	76.95	96
25% Percentile	5.21	14.91	23.36	25	0.83	22.35	27
Median	12.89	22.87	39.33	43	2.27	32.18	43
75% Percentile	23.36	39.05	59.38	61	4.62	43.63	58
Mean	16.98	26.9	43.88	44.88	3.82	35.31	43.43
Std. Deviation	15.32	17.16	23.34	22.20	8.521	15.59	20.16
Std. Error of Mean	1.60	1.79	2.43	2.31	0.89	3.25	4.20

**Table 2:** Stratification in ranges: <20 ng/mL, sub-therapeutic; 20–60 ng/mL, therapeutic range; >60, supra-therapeutic.

		Number of Samples (%)				Statistics		
		Alinity C				Cohen’s Weighted Kappa		
Active Moiety		<20 ng/mL	20–60 ng/mL	>60 ng/mL	Total	K	SE	CI 95%
**UHPLC-MS/MS**	<20 ng/mL	8 (8.7)	5 (5.43)	0	13	0.72	0.06	0.58–0.95
	20–60 ng/mL	2 (2.17)	51 (55.43)	5 (5.43)	58	**McNemar test**		
	>60 ng/mL	0	3 (3.26)	18 (19.57)	21	**(<20 ng/mL)**–**(20–60 ng/mL)**	**(20–60 ng/mL)**–**(>60 ng/mL)**	
	Total	10	59	23	92	*p*-value = 0.45	*p*-value = 0.72	

**Table 3:** Stratification in ranges: <20 ng/mL, sub-therapeutic; 20–60 ng/mL, therapeutic range; >60, supra-therapeutic.

		Number of Samples (%)				Statistics		
		Alinity C				Cohen’s Weighted Kappa		
Paliperidone		<20 ng/mL	20–60 ng/mL	>60 ng/mL	Total	K	SE	CI 95%
**UHPLC-MS/MS**	<20 ng/mL	1 (4.35)	2 (8.7)	0	3	0.63	0.18	0.26–1
	20–60 ng/mL	0	17 (73.91)	1 (4.35)	18	**McNemar test**		
	>60 ng/mL	0	0	2 (8.7)	2	**(<20 ng/mL)**–**(20–60 ng/mL)**	**(20–60 ng/mL)**–**(>60 ng/mL)**	
	Total	1	19	3	23	*p*-value = 0.5	*p*-value = 1	

**Table 4:** Mobile phase gradient.

Time (min)	Mobile Phase A (%)	Mobile Phase B (%)
0	85.0	15.0
0.5	85.0	15.0
3.00	30.0	70.0
4.00	5.0	95.0
4.01	85.0	15.0
6	85.0	15.0

## Data Availability

Dataset available on request from the authors.

## Supplementary Materials

The following supporting information can be downloaded at: https://www.mdpi.com/article/10.3390/ph17111446/s1, Figure S1. Bland-Altman’s plot. Percentage of difference in active moiety concentrations (risperidone plus paliperidone) vs. the average between Alinity C and UHPLC-MS/MS. Dashed green line represents the bias difference and dashed blue lines represent the limits of agreement. Risperidone therapeutic reference range is represented by dashed black lines. Figure S2. Bland-Altman’s plot. Percentage of difference in paliperidone concentrations vs the average between Alinity C and UHPLC-MS/MS. Dashed green line represents the bias difference and dashed blue lines represent the limits of agreement. Paliperidone therapeutic reference range is represented by dashed black lines.
