# Supplementary material for: Head-to-Head Comparison of UHPLC-MS/MS and Alinity C for Plasma Analysis of Risperidone and Paliperidone
Source: Pharmaceuticals (Basel). 2024 Oct 29;17(11):1446. doi: 10.3390/ph17111446 (PMC11597569; doi:10.3390/ph17111446)
Supplement: Supplementary file 1 [file pharmaceuticals-17-01446-s001.zip › pharmaceuticals-3264940-supplementary.pdf]

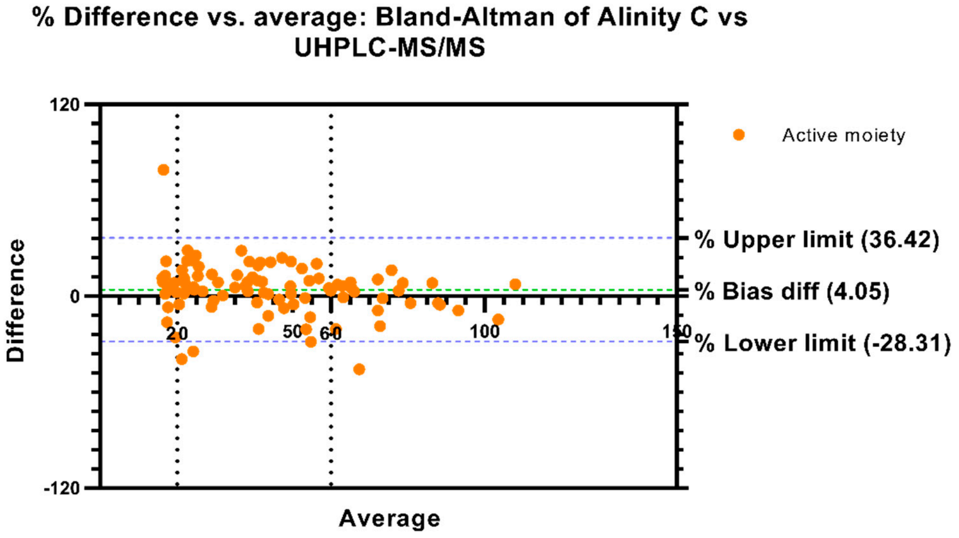

Figure S1. Bland-Altman's plot. Percentage of difference in active moiety concentrations (risperidone plus paliperidone) vs the average between Alinity C and UHPLC-MS/MS. Dashed green line represents the bias difference and dashed blue lines represent the limits of agreement. Risperidone therapeutic reference range is represented by dashed black lines.

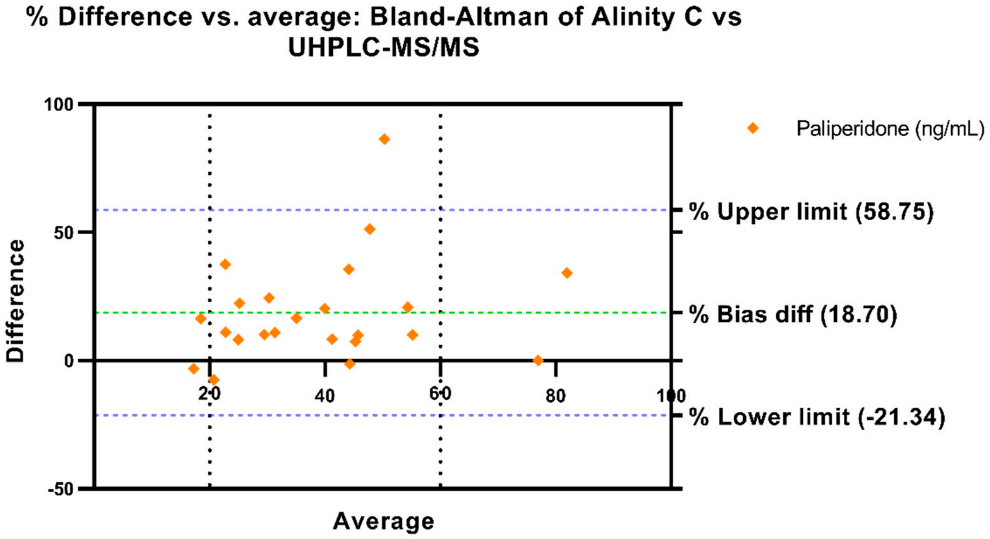

Figure S2. Bland-Altman's plot. Percentage of difference in paliperidone concentrations vs the average between Alinity C and UHPLC-MS/MS. Dashed green line represents the bias difference and dashed blue lines represent the limits of agreement. Paliperidone therapeutic reference range is represented by dashed black lines.
